# LncCE: Landscape of Cellularly-elevated lncRNAs in Single Cells Across Normal and Cancer Tissues

**DOI:** 10.1093/gpbjnl/qzaf069

**Published:** 2025-08-20

**Authors:** Kang Xu, Yujie Liu, Chongwen Lv, Ya Luo, Jingyi Shi, Haozhe Zou, Weiwei Zhou, Dezhong Lv, Changbo Yang, Yongsheng Li, Juan Xu

**Affiliations:** College of Bioinformatics Science and Technology, Harbin Medical University, Harbin 150081, China; College of Bioinformatics Science and Technology, Harbin Medical University, Harbin 150081, China; College of Bioinformatics Science and Technology, Harbin Medical University, Harbin 150081, China; College of Bioinformatics Science and Technology, Harbin Medical University, Harbin 150081, China; College of Bioinformatics Science and Technology, Harbin Medical University, Harbin 150081, China; College of Bioinformatics Science and Technology, Harbin Medical University, Harbin 150081, China; College of Bioinformatics Science and Technology, Harbin Medical University, Harbin 150081, China; College of Bioinformatics Science and Technology, Harbin Medical University, Harbin 150081, China; College of Bioinformatics Science and Technology, Harbin Medical University, Harbin 150081, China; School of Interdisciplinary Medicine and Engineering, Harbin Medical University, Harbin 150081, China; College of Bioinformatics Science and Technology, Harbin Medical University, Harbin 150081, China

**Keywords:** lncRNA, LncCE, Single-cell sequencing, Cancer, Cellularly-elevated

## Abstract

Long non-coding RNAs (lncRNAs) have emerged as significant players in maintaining the morphology and function of tissues and cells. The precise regulatory effectiveness of lncRNAs is closely associated with their spatial expression patterns across tissues and cells. Here, we propose the Cellularly-Elevated LncRNA (LncCE) resource to systematically explore cellularly-elevated (CE) lncRNAs across normal and cancer tissues at single-cell resolution. LncCE encompasses 87,946 entries of CE lncRNAs of 149 cell types by analyzing 181 single-cell RNA sequencing datasets, involving 20 fetal normal tissues, 59 adult normal tissues, 32 adult cancer types, and 5 pediatric cancer types. Two main search options are provided via a given lncRNA name or cell type. The results emphasize both qualitative and quantitative expression features of lncRNAs across different cell types, their co-expression with protein-coding genes, and their involvement in biological functions. In particular, LncCE provides quantitative visualizations of lncRNA expression changes in cancers compared to control samples, as well as clinical associations with patients’ overall survival. Together, LncCE offers an extensive, quantitative, and user-friendly interface to create a CE expression atlas for lncRNAs across normal and cancer tissues at the single-cell level. The LncCE database is available at http://bio-bigdata.hrbmu.edu.cn/LncCE.

## Graphical abstract

**Graphical Abstract:**
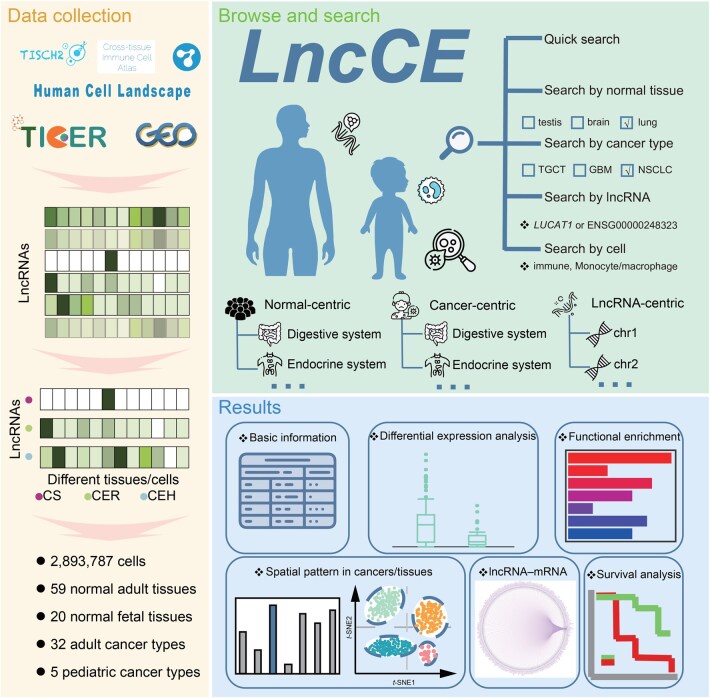

## Introduction

Long non-coding RNAs (lncRNAs) are a class of non-coding RNAs that are longer than 200 nt and usually not translated into proteins [1–3]. It is widely accepted that lncRNAs are closely associated with various crucial biological functions to maintain tissue morphology, and are also likely involved in cancer progression [4–6].

Emerging studies have comprehensively characterized the expression patterns of genes with bulk transcriptomes. Besides tissue-specific genes, two other kinds of genes have been found to be important for exertion of the physiological functions of normal tissues as well as initiation and progression of cancer, *i.e.*, tissue-enhanced and tissue-enriched genes [7,8]. These three categories of genes all exhibit tissue-elevated expression in certain tissues; that is, the expression of these genes is dominantly higher in several tissues than in other tissues. Genes with tissue-elevated expression patterns have been constantly identified and validated across normal and cancer tissues [9–11], and their biomarker potential for cancer diagnosis and prognosis has also been discussed [6,12]. Thus, comprehensive characterization of spatial expression can reveal the function of lncRNAs across tissues and cancers. Indeed, current developments in transcriptome analyses unveil stronger tissue-specific expression of lncRNAs than protein-coding genes [13]. In our previous studies, the tissue-elevated expression patterns of lncRNAs were systematically explored across normal and cancer tissues, with their key roles revealed in maintaining morphology and function of tissues [6,12].

Single-cell RNA sequencing (scRNA-seq) technologies are powerful for analyzing the spatial expression patterns of genes at the single-cell level [14], providing an unprecedented chance to highlight increasingly challenging biological questions and explore the molecular mechanisms related to carcinogenesis [15–17]. Emerging studies also have investigated the expression patterns of lncRNAs across normal and cancer cells [18–21], and further experimentally validated lncRNAs with cell state-specific functions involved in cell cycle progression and apoptosis [22,23]. For example, lncRNAs specially expressed in T cells have been found to play important roles in cancer immunity [24]. However, little is yet known about lncRNA expression properties at the single-cell level, and there is still no comprehensive database providing a cellularly-elevated (CE) lncRNA expression atlas across human tissues and cancers utilizing large-scale scRNA-seq data.

To address these challenges, we constructed the public resource, Cellularly-Elevated LncRNA database (LncCE; http://bio-bigdata.hrbmu.edu.cn/LncCE), which is a landscape of CE lncRNAs in single cells across normal and cancer tissues. The CE lncRNAs were comprehensively determined, and further classified into three categories based on their dominance in a certain cell type, including cell-specific (CS), cell-enriched (CER), and cell-enhanced (CEH). LncCE not only provides the CE expression patterns of lncRNAs but also can be used for downstream analysis, such as identifying novel cellular markers and comparing CE expression patterns of lncRNAs across different cellular states. LncCE may thus significantly help the research community understand the biological functions of lncRNAs in cells, tissues, and during tumorigenesis.

## Data collection and processing

### Data collection and pre-processing

For single-cell RNA (scRNA) transcriptome resources across normal tissues, three widely available transcriptome datasets were collected (Table S1), including the Human Cell Landscape [25], Cross-tissue Immune Cell Atlas [26], and The Tabula Sapiens [27]. In particular, one normal fetal transcriptome dataset, the Human Cell Landscape Fetal dataset, was also included in our study for a comprehensive comparison of CE lncRNAs during development.

For scRNA transcriptome resources across cancer types, we collected scRNA raw count files from Gene Expression Omnibus [28], ArrayExpress [29], Tumor Immune Single-cell Hub (TISCH) [30], and TISCH2 [31], complemented by datasets obtained directly from published studies [32]. Similarly, pediatric cancer transcriptome datasets were also collected, including five pediatric cancer types [33,34].

For all scRNA transcriptome resources, we also collected the metadata information (including sample ID, organ/tissue origin, clinical treatment, biosample group, and major cell types) for each normal or cancer tissue. For all scRNA datasets, we removed genes that were not expressed in at least three cells and cells that did not have at least 50 detectable genes. The data were then normalized using a scale factor of 10,000 and natural log-transformed. In total, LncCE documents 2,893,787 cells from 181 transcriptome resources, including 74 transcriptomes of 32 adult cancer types, 5 transcriptomes of 5 pediatric cancer types, 82 transcriptomes of 59 adult normal tissues, and 20 transcriptomes of 20 fetal normal tissues. LncCE uses the original major cell type annotation from the metadata information. To provide comprehensive and precise annotations of similar cell types, we corrected and unified the cell types; for example, in some datasets, the cell names were misspelled or the same cell type had different names in different datasets. Furthermore, to identify clusters of distinct cell populations, the *t*-distributed stochastic neighbor embedding (*t*-SNE) clustering algorithm was implemented in the Seurat R package (v4.3.0).

### Gene annotation

Gene annotation files were downloaded from GENCODE (release 38, GRCh38) [35] which includes different types of genes, including protein-coding genes, lncRNAs, and pseudogenes. For a more comprehensive analysis of lncRNA expression patterns at the single-cell level, we considered pseudogenes as lncRNAs in our study.

### CE lncRNA identification

To identify CE lncRNAs in various cells, we first selected lncRNAs with expression levels that were at least five-fold higher in a specific cell type compared to all other cell types [6,9,12,36]. These CE lncRNAs were then categorized into three distinct groups based on the degree of elevated expression in the particular cell: CS, CER, and CEH. CS lncRNAs are expressed exclusively in one specific cell type, with a minimum expression threshold of 0.001 counts per million. CER lncRNAs demonstrate at least a five-fold increase in expression in one cell type compared to the highest expression levels in any other cell type. Finally, CEH lncRNAs are those with at least five-fold higher expression levels in a particular cell type compared with the average expression levels in all other cell types. Additionally, CE lncRNAs are defined as those that expressed in more than ten cells of the same type. This methodology was also applied to identify CE lncRNAs in various cancer cell types. In a similar manner, CE protein-coding genes were identified for each cell type in both normal and cancerous conditions.

### lncRNA–mRNA correlation

scRNA-seq may not be able to detect the truly expressed gene in some cells and is therefore misrepresented by false zero expression. Given that scRNA-seq data may be much more sparse than bulk RNA sequencing (RNA-seq) data from whole tissues, we applied scLink, a new method to better characterize the statistical dependencies between genes in a single cell [37]. Users can set different correlation thresholds and spatial patterns of messenger RNA (mRNAs) to visualize the lncRNA–mRNA co-expression subnetworks based on the ECharts tool. Moreover, CE mRNAs in the same cell are also highlighted in the co-expression subnetwork by node colors.

### Function prediction of CE lncRNAs

Function prediction of CE lncRNAs is also provided at the single-cell level. After users select the co-expressed mRNAs in the previous step, the genes are subjected online to the R packages “clusterProfiler” to predict enriched functions of the lncRNAs, including Gene Ontology categories and pathways [38].

### Database construction

LncCE was constructed by Java Server Pages and deployed on Tomcat software (v6). All datasets in LncCE were documented and managed in MySQL database (v5.5). Several commonly used Java script packages, including ECharts (v5.2.2), DataTable (v1.12.1), Highcharts (v7.1.2), and Plotly (v2.16.1), were implemented for presentation of query results and interactive visualization of data. All data processing and integration analysis were performed using R software (v4.1.2). Currently, the website has been tested on several popular web browsers, including Google Chrome (preferred), Firefox, and Apple Safari browsers.

## Database content and usage

### Data summary

The current version of LncCE includes 2,893,787 cells, representing 149 cell types across 37 cancer types and 79 normal tissues in the human body. This database comprises 20 fetal normal tissues, 59 adult normal tissues, and 32 adult cancer types, as well as 5 pediatric cancer types. On average, each scRNA-seq dataset contains 15,988 cells, with a range from 133 to 103,703 cells (Table S1). A total of 14,941 lncRNAs have been identified in the LncCE database. The CE lncRNAs were comprehensively identified (details in Data collection and processing) and were further classified into three categories: CS lncRNAs, CER lncRNAs, and CEH lncRNAs. Currently, 87,946 entries of CE lncRNAs are curated in LncCE (Table S2), with the largest number of CE lncRNAs in the lacrimal gland functional unit cell of the eye (The Tabula Sapiens, *n* = 3342) and the smallest number of CE lncRNAs in a B cell from the breast cancer dataset (GSE114727_indrop, *n* = 1).

### Database overview

**Figure 1:**
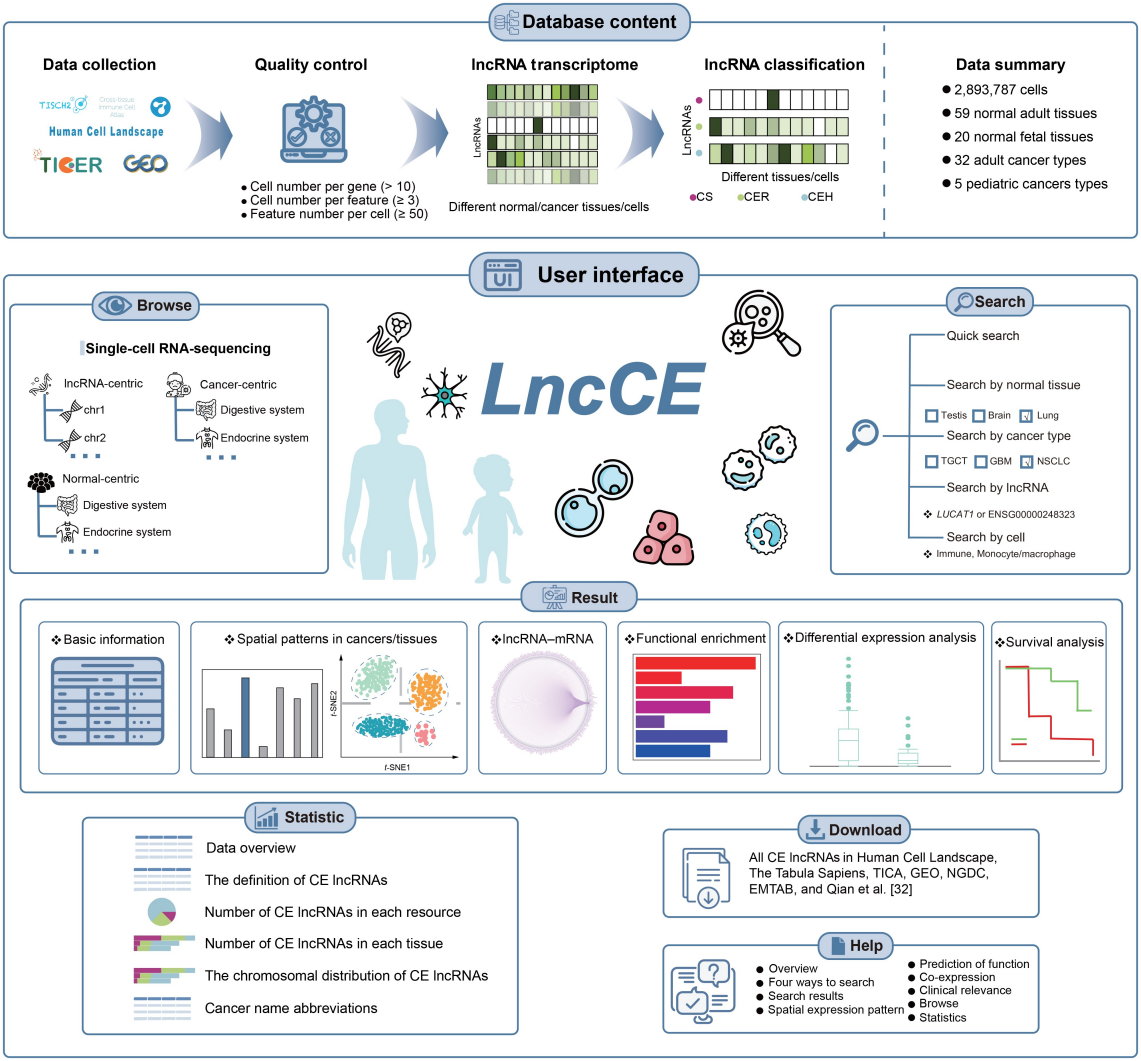
Schematic of the overall design of LncCE Top: workflow of obtaining all CE lncRNAs from multiple scRNA-seq datasets. Bottom: user interface of LncCE. The users can select adult or fetal normal tissues, or adult or pediatric cancer tissues for quick queries. In this panel, the Search, Browse, Statistic, Download, and Help modules provide flexible ways to access the dataset. Four types of search modules are provided for all CE lncRNAs, along with three methods for browsing. CE, cellularly-elevated; lncRNA, long non-coding RNA; LncCE, Cellularly-Elevated LncRNA; scRNA-seq, single-cell RNA sequencing; CS, cell-specific; CER, cell-enriched; CEH, cell-enhanced; TGCT, testicular germ cell tumor; GBM, glioblastoma; NSCLC, non-small cell lung cancer; mRNA, messenger RNA; TICA, Tissue-immune cell atlas; GEO, Gene Expression Omnibus; NGDC, National Genomics Data Center; EMTAB, Experiment – Microarray/Transcriptomics ArrayExpress B.

LncCE is not only a comprehensive resource for CE lncRNAs across single-cell analyses but also offers a user-friendly web interface for investigating the spatial expression of lncRNAs across various cellular states (**Figure 1**). Users can easily toggle between adult and pediatric normal/cancer tissues by clicking the appropriate button on the “Home” page. From the “Home” page, users can navigate to the “Browse”, “Search”, and “Download” pages to browse, search for, and download all CE lncRNAs available in LncCE.

CE lncRNAs are organized into three browsing categories: “lncRNA-centric”, “Normal-centric”, and “Cancer-centric”. In the “lncRNA-centric” section, all CE lncRNAs are displayed in a hierarchical structure based on their chromosomal localization. The “Normal-centric” and “Cancer-centric” sections categorize adult/fetal normal tissues and adult/pediatric cancer tissues according to their anatomical classification in the human body map, respectively. Additionally, users can quickly access the “Searching Result” pages by clicking directly on the tissue of interest displayed on the “Home” page. The “Search” sections offer four different query options, allowing users to filter based on the type of tissue (normal or cancer), lncRNA name, or cell type. Statistical data regarding the CE lncRNAs in LncCE are available on the “Statistic” page, and users can download all the data freely from the “Download” page. For detailed guidance on how to navigate and query the data, users can refer to the tutorial provided on the “Help” page.

### lncRNA-based exploration with LncCE

Recently, several studies have indicated that lung cancer associated transcript 1 (*LUCAT1*) is a cancer-related and myeloid cell-specific lncRNA that can interact with signal transducer and activator of transcription 1 (*STAT1*) to inhibit the transcription of interferon-stimulated genes [39–41]. As a practical example of using the “lncRNA” search feature, we utilized LncCE to identify the cell types expressing *LUCAT1* across various tissues.

**Figure 2:**
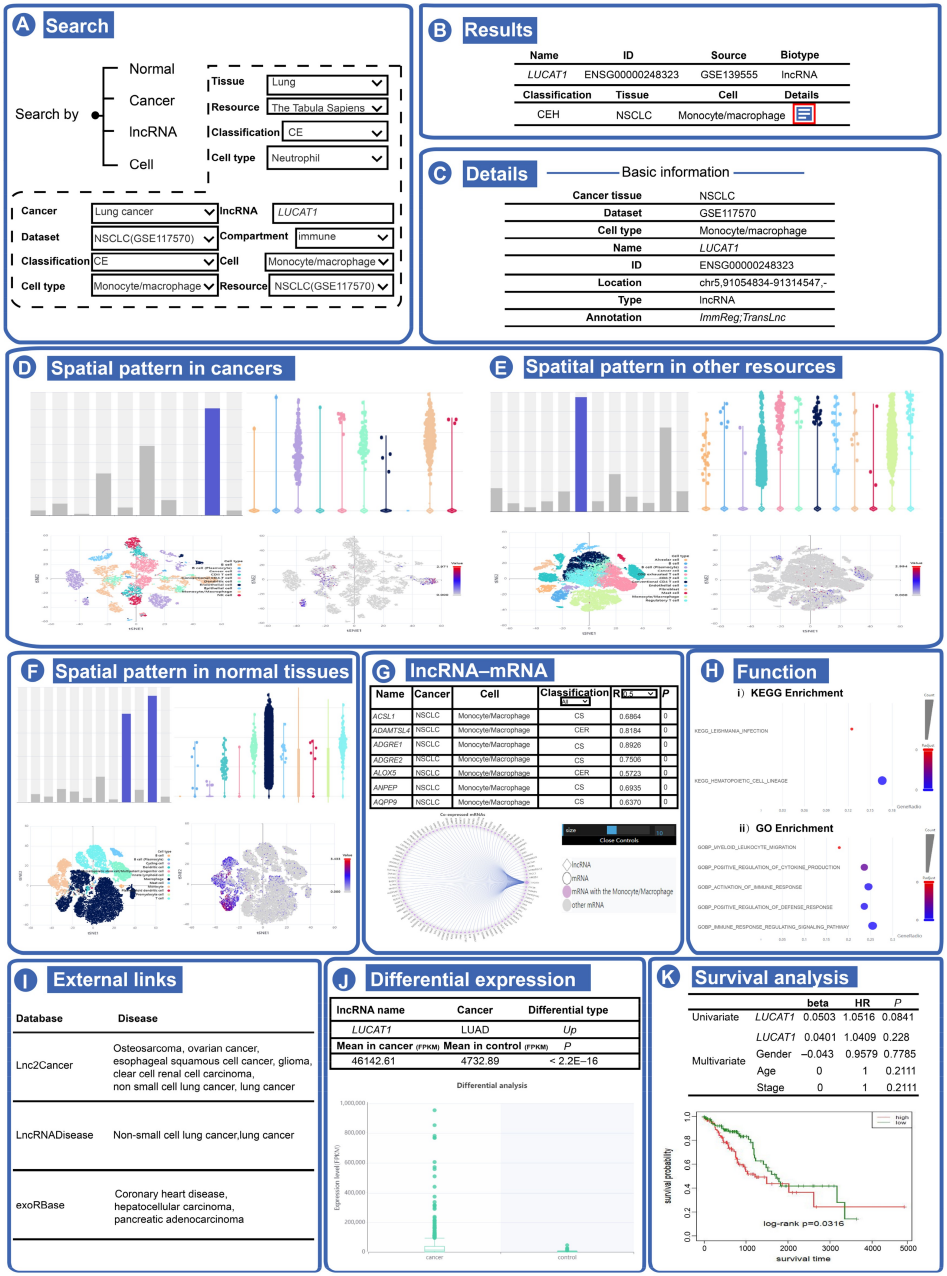
lncRNA-based exploration with LncCE **A**. Search by adult or fetal normal tissues, adult or pediatric cancer tissues, lncRNAs, or cell types of interest. **B**. The result list for lncRNAs. **C**. Basic annotation information for CE lncRNA *LUCAT1* and annotations in other relevant databases. **D**. A global map of different cell populations and expression levels of *LUCAT1* across cell types in CE cancer tissue (NSCLC) in the selected dataset (GSE117570). **E**. A global map of different cell populations and expression levels of *LUCAT1* across cell types in CE cancer tissue (NSCLC) in other datasets. **F**. A global map of different cell populations and expression levels of *LUCAT1* across cell types in normal tissues (lung) or those associated with CE cancer tissue (NSCLC). **G**. Co-expression network between CE lncRNA *LUCAT1* and mRNAs. **H**. Functional and pathway enrichment analyses of co-expressed mRNAs. **I**. External links related to NSCLC and *LUCAT1*. **J**. Expression and regulation of lncRNA *LUCAT1* in TCGA cancer specimens. **K**. Survival analysis of CE lncRNA *LUCAT1* in TCGA cancer specimens. TCGA, The Cancer Genome Atlas.

Upon entering a lncRNA in the designated search field within the “lncRNA” section of the “Search” page, the LncCE displays a table containing the gene’s basic information for each cell type across all datasets where the gene is identified as a CE lncRNA (**Figure 2**A and B). Our search for *LUCAT1* reveals that it is predominantly expressed in myeloid cells in most cancers including lung cancer and colorectal cancer, and is also highly expressed in myeloid cells in normal tissues including lung and esophagus tissues (Figure 2A).

By clicking the details button in these tables, users can access additional information for each entry. A hyperlink directs users to the detailed result page for the CE lncRNA *LUCAT1* in non-small cell lung cancer (NSCLC), derived from GSE117570. This page offers eight key types of information (Figure 2C–K): (1) basic annotation information, with links to resources such as ImmReg [42], TransLnc [43], and LncSpA [6] for further *LUCAT1* annotations; (2) a table presenting CE cancer tissue (NSCLC), CE subclassifications, and related expression levels, with visuals including bar and box plots for expression across different cell types, along with a *t*-SNE plot colored by cell type to illustrate lncRNA expression (*e.g.*, *LUCAT1*) across cell types; (3) both qualitative and quantitative spatial expression patterns in normal tissues, with lung tissue as the CE tissue; (4) a network view displaying co-expression between CE lncRNA *LUCAT1* and mRNAs, with correlation data in an accompanying table, where users can adjust thresholds (0–0.7) to filter the lncRNA–mRNA co-expression network; (5) functional and pathway enrichment analyses of co-expressed mRNAs highlighting connections to physiological and pathological lung tissue states; (6) evidence linking NSCLC and *LUCAT1* from sources such as Lnc2Cancer [44], LncRNADisease [45], and exoRBase [46]; (7) evidence from The Cancer Genome Atlas (TCGA) indicating that expression of *LUCAT1* in cancer is upregulated in lung adenocarcinoma and lung squamous cell carcinoma, supporting its potential role as an oncogenic lncRNA, consistent with the findings from a previous study [39]; and (8) regression analysis and the Kaplan–Meier survival plot indicating that *LUCAT1* acts as a protective factor in lung adenocarcinoma and lung squamous cell carcinoma. Together, these eight sections provide a comprehensive view for understanding CE lncRNA functionality across different cell types in both normal and cancer tissues.

### Cell type-based exploration with LncCE

Emerging studies have thoroughly characterized the functions of cell type-elevated mRNAs in both normal and cancer tissues [47–49], but research on lncRNAs has been comparatively limited. LncCE provides a comprehensive annotation of cell type-elevated lncRNAs, enabling investigation into the functions of these CE lncRNAs. These lncRNAs are specifically expressed in a variety of cell types (Table S3), including immune cells (such as T cells, B cells, and macrophages), stromal cells, endothelial cells, and muscle cells.

**Figure 3:**
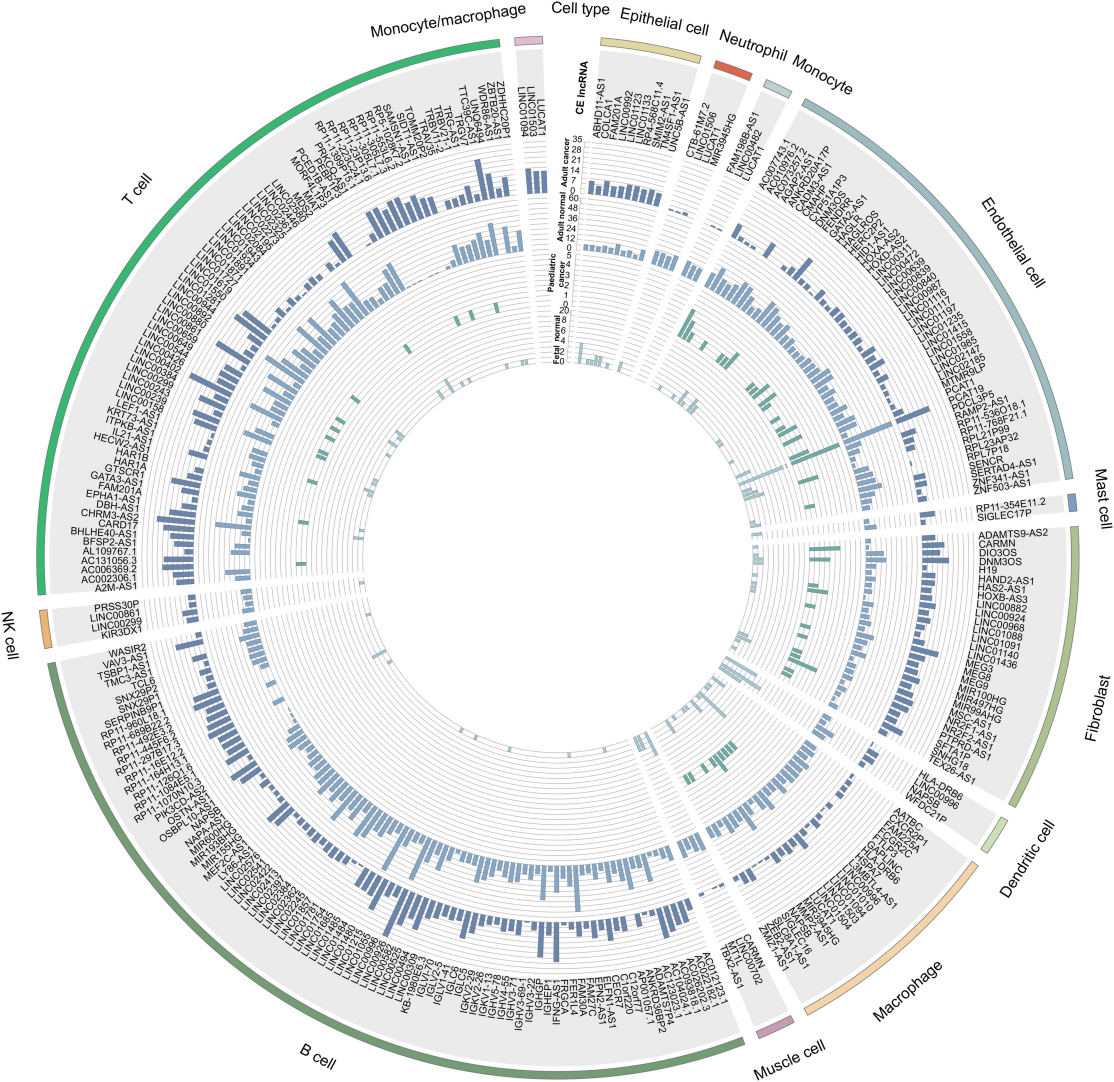
Distribution of CE lncRNAs in each cell type across adult and fetal normal tissues as well as adult and pediatric cancer tissues The number of CE lncRNAs in each cell type is more than 14. The outermost circle represents cell types, with the arc length proportional to the quantity of CE lncRNAs. The penultimate circle contains the names of CE lncRNAs. The third circle shows the distribution of CE lncRNA counts in adult cancer tissues, followed by adult normal tissues, pediatric cancer tissues, and fetal normal tissues. The height of each bar corresponds to the number of datasets in which the lncRNA is identified as a CE lncRNA. NK, natural killer.

**Figure 4:**
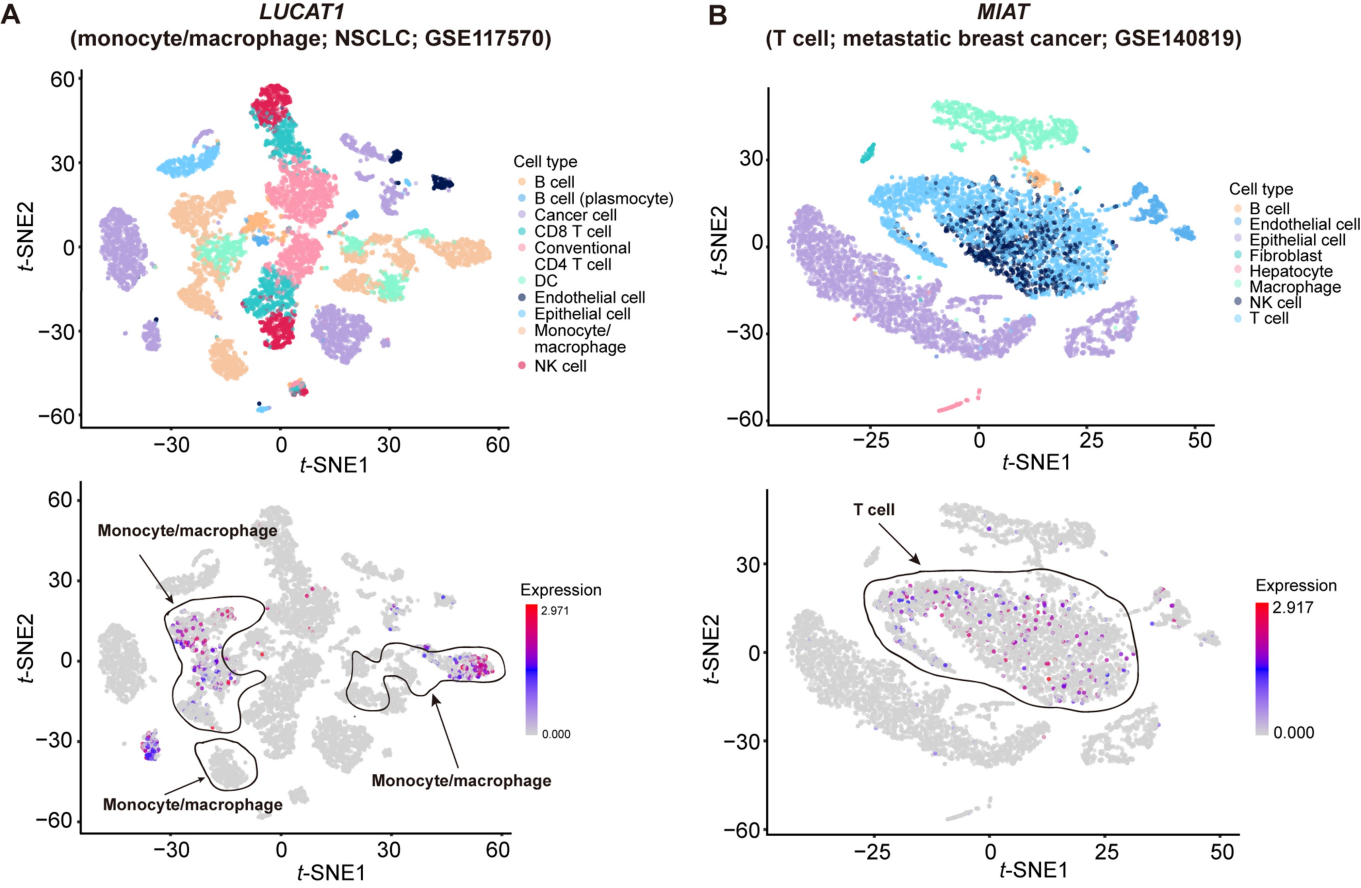
Cell type-based exploration with LncCE **A**. *t*-SNE plots of the GSE117570_NSCLC dataset, colored by cell type (top) and *LUCAT1* expression (bottom). **B**. *t*-SNE plots of the GSE140819_Metastatic_breast_cancer dataset, colored by cell type (top) and *MIAT* expression (bottom). *t*-SNE, *t*-distributed stochastic neighbor embedding.

Next, we focused on CE lncRNAs identified in at least 15 datasets (**Figure 3**). Several lncRNAs, including *LUCAT1*, myocardial infarction associated transcript (*MIAT*), WAP four-disulfide core domain 21 (*WFDC21P*), cardiac mesoderm enhancer-associated non-coding RNA (*CARMN*), and prostate cancer associated transcript 19 (*PCAT19*), exhibit expression in matched cell types, suggesting a conserved role in these cell types. *LUCAT1* is reported as a myeloid-specific lncRNA, and it is identified as a CE lncRNA in 85 datasets in LncCE (**Figure 4**A), particularly in myeloid derived cells [macrophages, monocytes, neutrophils (66/85), and myeloid cells (7/85)]. *MIAT* serves as a T cell marker in LncCE, which is identified as a CE lncRNA in 20 adult cancer datasets (Figure 4B), and it is primarily expressed in tumor and T cells, indicating that *MIAT* may be involved in the immune escape process of cancer [50]. *WFDC21P*, also known as *lnc-DC*, is exclusively expressed in human dendritic cells and is essential for optimal dendritic cell differentiation from human monocytes. In addition, *WFDC21P* has been shown to interact with the transcription factor *STAT3* and promote its nuclear translocation and function [51], and has also been identified as a CE lncRNA of dendritic cells across 23 datasets (Figure S1A). *CARMN* has been reported as an evolutionarily conserved smooth muscle cell-specific lncRNA [52], and is identified as a CE lncRNA in endothelial and muscle cells (Figure S1B). *PCAT19* has been reported to safeguard DNA in quiescent endothelial cells by preventing uncontrolled phosphorylation of replication protein A2 (RPA2) [53], is implicated in driving prostate cancer [54], and has served as a prognostic biomarker for endometrial cancer [55]. In LncCE, *PCAT19* is identified as a CE lncRNA in 126 datasets, particularly in endothelial cells (108/126), suggesting that *PCAT19* may be a novel biomarker for endothelial cells (Figure S1C). These findings suggest that a considerable number of CE lncRNAs are involved in cellular differentiation, activation, and inflammation-based signaling, cancer initiation, development, and treatment resistance.

## Discussion

LncCE offers a comprehensive platform for examining the spatial expression patterns of lncRNAs at the single-cell level across adult and fetal normal tissues, as well as adult and pediatric cancer types. Its intuitive interface enables users to easily query, browse, and download CE lncRNAs of interest. The platform provides eight key types of information to facilitate visualization and a deeper understanding of the roles of CE lncRNAs in both physiological and pathological contexts.

Unlike other resources, LncCE is uniquely focused on CE lncRNAs across normal and cancer single-cell transcriptomic datasets (Table S4). In addition to offering spatial expression patterns of CE lncRNAs, LncCE supports various downstream analyses, including the discovery of novel cellular markers and comparison of spatial lncRNA patterns across different conditions. Therefore, LncCE provides four key applications for advancing research on CE lncRNAs (File S1). These applications include: (1) cell type-specific enrichment patterns of CE lncRNAs among tissues and cancers; (2) investigation of CE lncRNAs in tissues of the same origin and their cancerous counterparts; (3) exploration of cell type similarity across datasets; and (4) utilization of CE lncRNAs as cell markers. Future updates will expand the database to incorporate more cell types from both healthy and diseased tissues, ensuring that LncCE remains an essential resource. Additionally, drug susceptibility data for CE lncRNAs will be added to enhance its utility. We are confident that the LncCE database will become an invaluable resource for both experimental and computational researchers, helping close the knowledge gap between lncRNA expression and phenotypic outcomes.

## Data availability

The online database LncCE is publicly available at http://bio-bigdata.hrbmu.edu.cn/LncCE. It has been submitted to Database Commons [56] at the National Genomics Data Center (NGDC), China National Center for Bioinformation (CNCB), which is publicly accessible at https://ngdc.cncb.ac.cn/databasecommons/database/id/10224.

## Supplementary Material

qzaf069_Supplementary_Data

## CRediT author statement

**Kang Xu:** Conceptualization, Methodology, Validation, Investigation, Formal analysis, Data curation, Resources, Writing – original draft, Writing – review & editing, Visualization. **Yujie Liu:** Software, Methodology, Writing – original draft, Writing – review & editing, Visualization. **Chongwen Lv:** Data curation, Investigation, Writing – original draft, Writing – review & editing, Visualization. **Ya Luo:** Data curation, Visualization. **Jingyi Shi:** Resources, Visualization. **Haozhe Zou:** Data curation, Resources. **Weiwei Zhou:** Data curation, Resources. **Dezhong Lv:** Software, Data curation. **Changbo Yang:** Visualization. **Yongsheng Li:** Conceptualization, Methodology, Funding acquisition, Writing – review & editing, Supervision. **Juan Xu:** Conceptualization, Methodology, Funding acquisition, Writing – review & editing, Supervision. All authors have read and approved the final manuscript.
