# Supplementary material for: LncCE: Landscape of Cellularly-elevated lncRNAs in Single Cells Across Normal and Cancer Tissues
Source: Genomics Proteomics Bioinformatics. 2025 Aug 20;23(4):qzaf069. doi: 10.1093/gpbjnl/qzaf069 (PMC12558386; doi:10.1093/gpbjnl/qzaf069)
Supplement: qzaf069_Supplementary_Data [file qzaf069_supplementary_data.zip › File S1.docx]

**Applications based on LncCE resource**

**Application 1: cell type-specific enrichment patterns of CE lncRNAs among tissues and cancers**

Our analysis of cell type-specific enrichment patterns of cellularly-elevated long non-coding RNAs (CE lncRNAs) (Figure S2) revealed striking heterogeneity across biological contexts. Notably, cancer cell, epithelial cell, T cell, and B cell exhibited the highest enrichment of CE lncRNAs. In adult cancers, cancer cell and epithelial cell were most enriched, potentially reflecting their roles in tumorigenesis and epithelial–mesenchymal transition (EMT), a hallmark of cancer progression [1]. Adult normal tissues showed predominant enrichment in T cell and B cell, suggesting CE lncRNAs may regulate immune surveillance or lymphocyte differentiation. Pediatric cancers, however, displayed enrichment in muscle cell and neuroendocrine cell. Fetal normal tissues exhibited CE lncRNA enrichment in epithelial and endothelial cell, possibly linked to tissue morphogenesis and vascular development. This heterogeneity underscores CE lncRNAs as dynamic regulators of cell identity and disease mechanisms, offering novel targets for context-specific therapeutic interventions.

**Application 2: investigating CE lncRNAs in tissues of the same origin and their cancerous counterparts**

To investigate the differences in CE lncRNAs between normal brain tissues and glioma patients, we first quantified cancer-specific, normal-specific, and shared CE lncRNAs across matched cell types. Intriguingly, astrocytes harbored a higher proportion of normal tissue-specific CE lncRNAs, whereas oligodendrocytes exhibited a predominance of cancer-specific CE lncRNAs (Figure S3A). Leveraging the lncRNA–mRNA (messenger RNA) correlation data from the LncCE database, we further functionally characterized these lncRNA categories (Figure S3B).

In astrocytes, cancer-specific CE lncRNAs were enriched in cilia-related functions (*e.g.*, cilium movement and epithelial cilium movement involved in extracellular fluid movement). Shared and normal-specific CE lncRNAs predominantly associated with metabolic and transport processes (*e.g.*, chitin metabolic process and D-amino acid transport), suggesting roles in maintaining astrocyte homeostasis, which may be subverted during tumorigenesis [2].

In oligodendrocytes, all three categories (cancer-specific, shared, and normal-specific CE lncRNAs) were enriched in oligodendrocyte development pathways (*e.g.*, development and differentiation). This highlights their conserved developmental roles, even in malignant contexts, and implies potential hijacking of differentiation programs in glioma progression.

**Application 3: exploration of cell type similarity across datasets**

To investigate cell type similarity across independent datasets, we calculated the Jaccard similarity of CE lncRNA profiles between cell types from different datasets (Figure S4). Hierarchical clustering of all datasets grouped cells into 30 distinct clusters. Strikingly, biologically related cell types consistently co-clustered. Cluster 4 comprised B cell and plasmocyte, reflecting their shared lymphoid lineage differentiation. Cluster 9 aggregated stromal cell (fibroblast, smooth muscle cell, and pericyte), consistent with their coordinated roles in extracellular matrix remodeling and vascular support. Cluster 2 contained natural killer (NK) cell and T cell, aligning with their functional synergy in adaptive immunity. Cluster 3 included myeloid cell, macrophage, and monocyte, mirroring their common hematopoietic origin and phagocytic functions. This conserved clustering pattern across diverse datasets demonstrates the robustness of CE lncRNAs as cell type-specific signatures.

**Application 4: CE lncRNAs utility as cell markers**

To evaluate the potential of CE lncRNAs as cell type-specific markers, we intersected CE lncRNAs from our database with lncRNA markers cataloged in the CellMarker database [3]. Notably, 478 CE lncRNAs in our resource were validated in CellMarker (Figure S5A), including well-characterized markers such as nuclear paraspeckle assembly transcript 1 (*NEAT1*) (Figure S5B and C) [4–6], maternally expressed 3 (*MEG3*) (Figure S5D) [5,7], lung cancer associated transcript 1 (*LUCAT1*) (Figure 2) [8,9], and WAP four-disulfide core domain 21 (*WFDC21P*) (Figure S1A) [10–12].

Intriguingly, our database uniquely identified additional lncRNAs with emerging roles as cell type markers, such as cardiac mesoderm enhancer-associated non-coding RNA (*CARMN*) (Figure S1B) — a conserved lncRNA essential for smooth muscle cell differentiation and endothelial function [13,14] — which is absent in CellMarker. This highlights LncCE’s ability to capture novel, functionally relevant markers for understudied cell types that are overlooked in existing resources. These results position CE lncRNAs as robust biomarkers for cell identity annotation.

**References**

[1] Castaneda M, den Hollander P, Kuburich NA, Rosen JM, Mani SA. Mechanisms of cancer metastasis. Semin Cancer Biol 2022;87:17–31.

[2] Perelroizen R, Philosof B, Budick-Harmelin N, Chernobylsky T, Ron A, Katzir R, et al. Astrocyte immunometabolic regulation of the tumour microenvironment drives glioblastoma pathogenicity. Brain 2022;145:3288–307.

[3] Hu C, Li T, Xu Y, Zhang X, Li F, Bai J, et al. CellMarker 2.0: an updated database of manually curated cell markers in human/mouse and web tools based on scRNA-seq data. Nucleic Acids Res 2023;51:D870–6.

[4] Villani AC, Satija R, Reynolds G, Sarkizova S, Shekhar K, Fletcher J, et al. Single-cell RNA-seq reveals new types of human blood dendritic cells, monocytes, and progenitors. Science 2017;356:eaah4573.

[5] Zhong S, Zhang S, Fan X, Wu Q, Yan L, Dong J, et al. A single-cell RNA-seq survey of the developmental landscape of the human prefrontal cortex. Nature 2018;555:524–8.

[6] Evren E, Ringqvist E, Tripathi KP, Sleiers N, Rives IC, Alisjahbana A, et al. Distinct developmental pathways from blood monocytes generate human lung macrophage diversity. Immunity 2021;54:259–75.e7.

[7] Li L, Dong J, Yan L, Yong J, Liu X, Hu Y, et al. Single-cell RNA-seq analysis maps development of human germline cells and gonadal niche interactions. Cell Stem Cell 2017;20:858–73.e4.

[8] Young MD, Mitchell TJ, Vieira Braga FA, Tran MGB, Stewart BJ, Ferdinand JR, et al. Single-cell transcriptomes from human kidneys reveal the cellular identity of renal tumors. Science 2018;361:594–9.

[9] Agarwal S, Vierbuchen T, Ghosh S, Chan J, Jiang ZZ, Kandasamy RK, et al. The long non-coding RNA *LUCAT1* is a negative feedback regulator of interferon responses in humans. Nat Commun 2020;11:6348.

[10] Meylan M, Petitprez F, Lacroix L, Di Tommaso L, Roncalli M, Bougouin A, et al. Early hepatic lesions display immature tertiary lymphoid structures and show elevated expression of immune inhibitory and immunosuppressive molecules. Clin Cancer Res 2020;26:4381–9.

[11] Petitprez F, de Reynies A, Keung EZ, Chen TW, Sun CM, Calderaro J, et al. B cells are associated with survival and immunotherapy response in sarcoma. Nature 2020;577:556–60.

[12] Wang P, Xue Y, Han Y, Lin L, Wu C, Xu S, et al. The STAT3-binding long noncoding RNA lnc-DC controls human dendritic cell differentiation. Science 2014;344:310–3.

[13] Dong K, Shen J, He X, Hu G, Wang L, Osman I, et al. *CARMN* is an evolutionarily conserved smooth muscle cell-specific lncRNA that maintains contractile phenotype by binding myocardin. Circulation 2021;144:1856–75.

[14] Ni H, Haemmig S, Deng Y, Chen J, Simion V, Yang D, et al. A smooth muscle cell-enriched long noncoding RNA regulates cell plasticity and atherosclerosis by interacting with serum response factor. Arterioscler Thromb Vasc Biol 2021;41:2399–416.
