# Supplementary material for: LncCE: Landscape of Cellularly-elevated lncRNAs in Single Cells Across Normal and Cancer Tissues
Source: Genomics Proteomics Bioinformatics. 2025 Aug 20;23(4):qzaf069. doi: 10.1093/gpbjnl/qzaf069 (PMC12558386; doi:10.1093/gpbjnl/qzaf069)
Supplement: qzaf069_Supplementary_Data [file qzaf069_supplementary_data.zip › supplementary material captions.docx]

**Supplementary material**

**File S1 Applications based on LncCE resource**

**Figure S1 Additional examples of cell type-based exploration with LncCE**

**A.** *t*-SNE plots of the GSE140228_LIHC dataset, colored by cell type and *WFDC21P* expression. **B.** *t*-SNE plots of the GSE139829_UVM dataset, colored by cell type and *CARMN* expression**. C.** *t*-SNE plots of the CRA001160_PAAD dataset, colored by cell type and *PCAT19* expression. t-SNE, t-distributed stochastic neighbor embedding. LIHC, liver hepatocellular carcinoma; UVM, uveal melanoma; PAAD, pancreatic ductal adenocarcinoma.

**Figure S2 Cell type-specific enrichment patterns of CE lncRNAs**

**Figure S3 Functional exploration of CE lncRNAs in glioma and normal brain tissues**

**A.** Cancer-specific, shared and normal-specific CE lncRNAs between glioma and brain in astrocyte and oligodendrocyte. **B.** Functional enrichment analysis of mRNAs associated with cancer-specific, shared, and normal-specific CE lncRNAs. P, positive; N, negative; HCL, human cell landscape.

**Figure S4 Cell type similarity across datasets based on CE lncRNAs**

**Figure S5 CE lncRNAs utility as cell markers**

**A.** The number of intersections in CellMarker and LncCE. **B.** Table of results based on *NEAT1* queries. **C.** *t*-SNE plots of the fetal brain in HCL dataset, colored by cell type and *NEAT1* expression. **D.** *t*-SNE plots of the BRCA in Qian et al., Cell Research 2020 dataset, colored by cell type and *MEG3* expression. BRCA, breast cancer.

**Table S1 The information of all datasets**

**Table S2 The number of CE lncRNAs of each dataset across adult/pediatric cancer/normal tissues**

**Table S3 The list of CE lncRNAs for each cell type**

**Table S4 Highlights of LncCE compared with other human scRNA-seq databases**
