# Supplementary figures and images for: LncCE: Landscape of Cellularly-elevated lncRNAs in Single Cells Across Normal and Cancer Tissues
Source: Genomics Proteomics Bioinformatics. 2025 Aug 20;23(4):qzaf069. doi: 10.1093/gpbjnl/qzaf069 (PMC12558386; doi:10.1093/gpbjnl/qzaf069)

## Slide 1
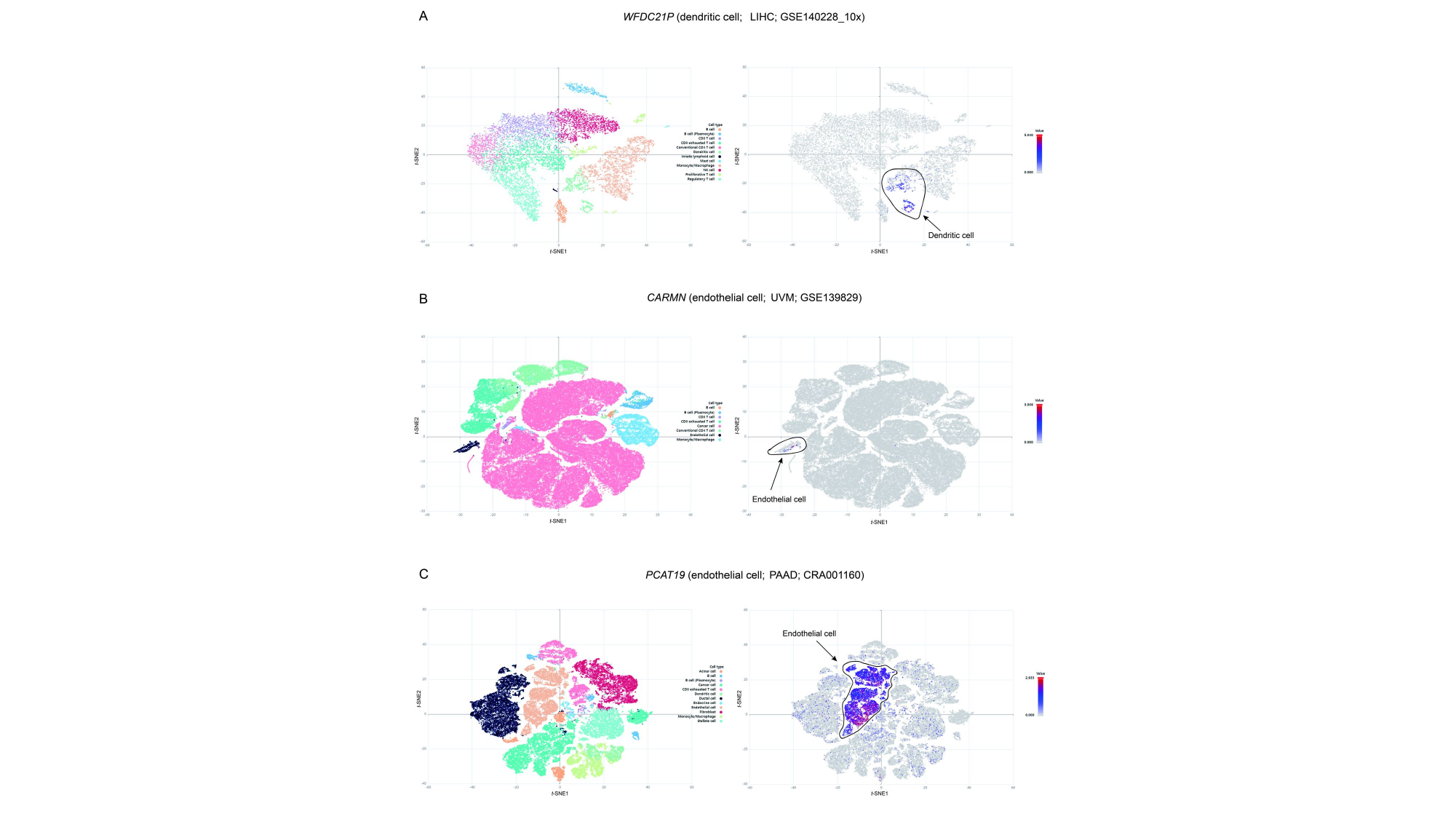

#

Supplement: qzaf069_Supplementary_Data [file qzaf069_supplementary_data.zip › Figure S1.pptx]

## Slide 1
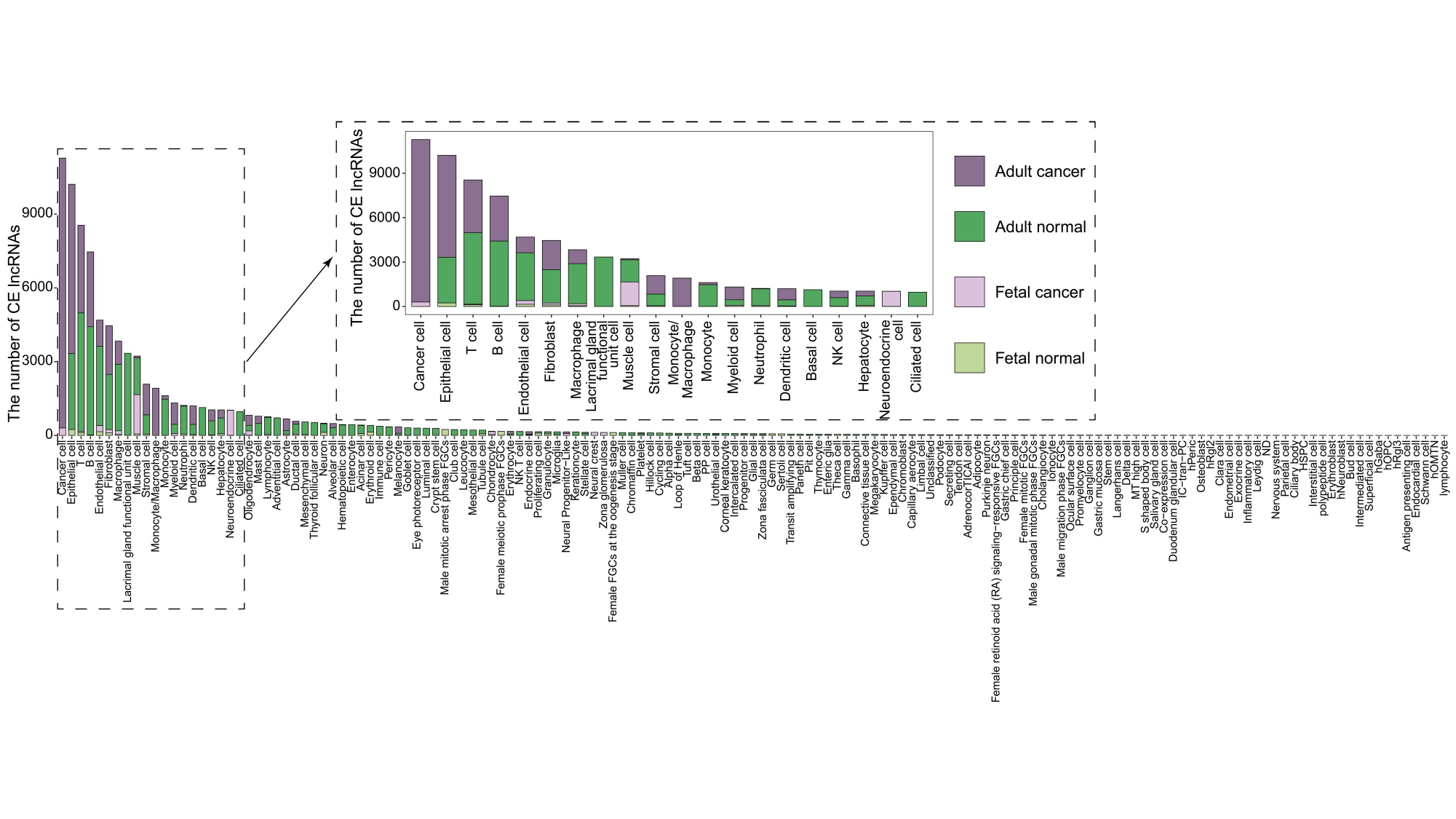

Supplement: qzaf069_Supplementary_Data [file qzaf069_supplementary_data.zip › Figure S2.pptx]

## Slide 1
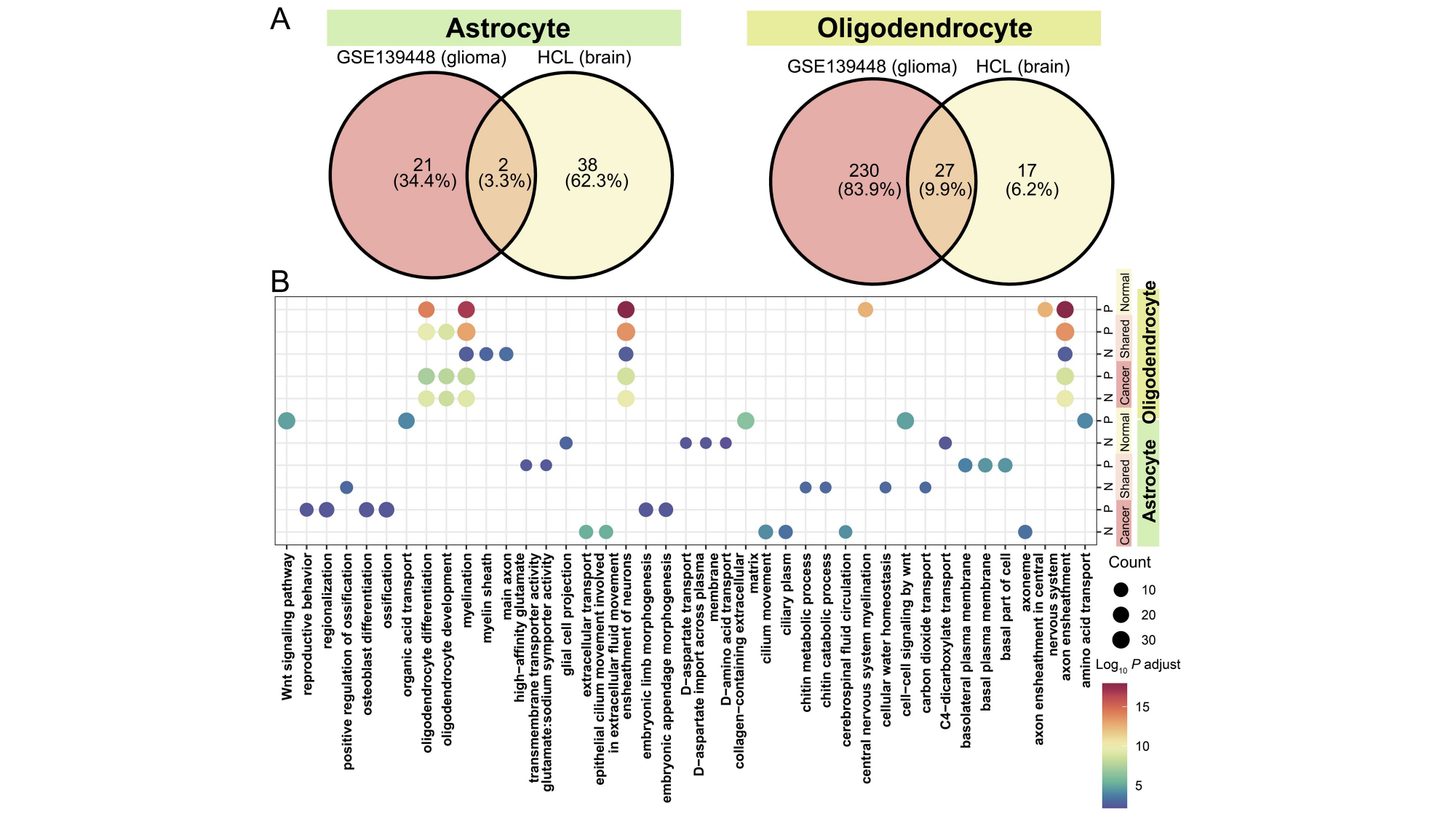

#

Supplement: qzaf069_Supplementary_Data [file qzaf069_supplementary_data.zip › Figure S3.pptx]

## Slide 1
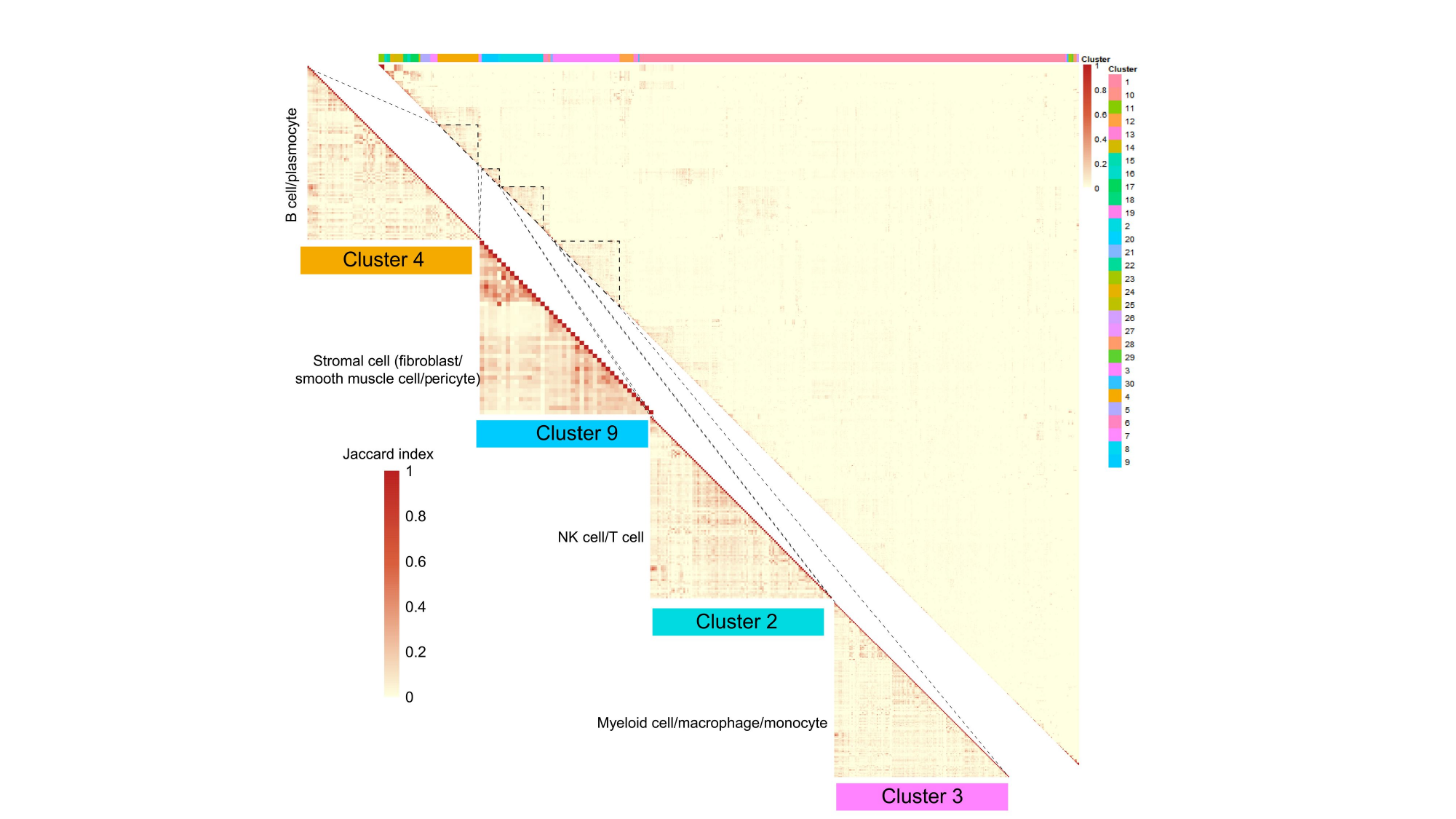

#

Supplement: qzaf069_Supplementary_Data [file qzaf069_supplementary_data.zip › Figure S4.pptx]

## Slide 1
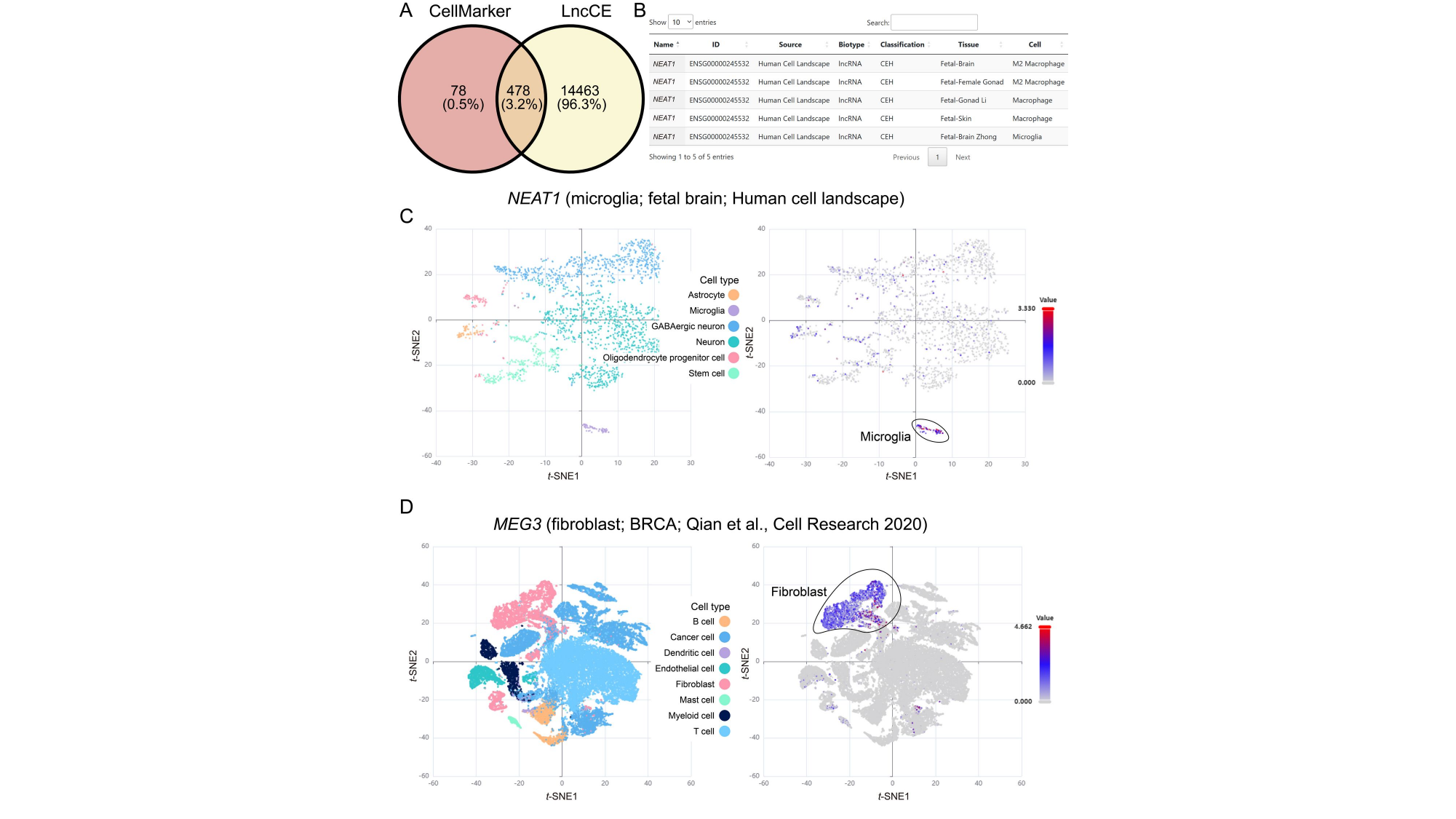

#

Supplement: qzaf069_Supplementary_Data [file qzaf069_supplementary_data.zip › Figure S5.pptx]
